# Comparing AI agents and machine learning for predicting mechanical and rheological properties of dense food structures

**DOI:** 10.1016/j.crfs.2026.101578

**Published:** 2026-09-21

**Authors:** Yizhou Ma

**Affiliations:** a Food Quality and Design, Wageningen University & Research, Wageningen, the Netherlands; b Food Process Engineering, Wageningen University & Research, Wageningen, the Netherlands

## Abstract

Food functionality prediction remains challenging because food materials are multicomponent, multiscale, and process-dependent. This challenge is particularly evident in plant-based meat analogues, in which composition, ingredient source, hydration, and processing history jointly determine structure and texture. This study compares three prediction paradigms for food mechanical measurements, namely deterministic machine learning (ML), inverse-distance-weighted (IDW) analog retrieval, and a knowledge-based AI agent that combined retrieval with explicit mechanistic priors. Two case studies were used. Case 1 involved out-of-distribution prediction of hardness and chewiness in plant-based meat analogues from proximate composition features. Case 2 involved prediction of storage modulus ($G^{\prime }$) and maximum stress at 200% strain in plant protein-polysaccharide mixtures. Across both cases, the knowledge-based agent matched or exceeded the ML baseline. In the plant-based meat analogue case, the agent achieved the best performance for hardness (MAE = 4.66 N, $R^{2}$ = 0.782) and near-best performance for chewiness (MAE = 4.45 J, $R^{2}$ = 0.696). In the rheology case, the agent achieved the best performance for both $G^{\prime }$ (MAE = 152.30 kPa, $R^{2}$ = 0.695) and maximum stress at 200% strain (MAE = 17.21 kPa, $R^{2}$ = 0.765). Pure IDW retrieval performed consistently worse, indicating that the main predictive gain came from mechanistic knowledge rather than retrieval alone. These results demonstrate that explicit domain priors can improve food functionality prediction, especially under distributional shift and for targets whose mechanisms are only partly encoded in numeric features.

## Highlights

• Knowledge-based agent outperforms machine learning in predicting mechanical properties.
• Mechanistic knowledge improves prediction accuracy under distribution shift.
• Pure similarity retrieval performs poorly without knowledge reasoning.
• Hybrid of data retrieval and reasoning enables robust small-data prediction.

## Graphical abstract

**Figure:**
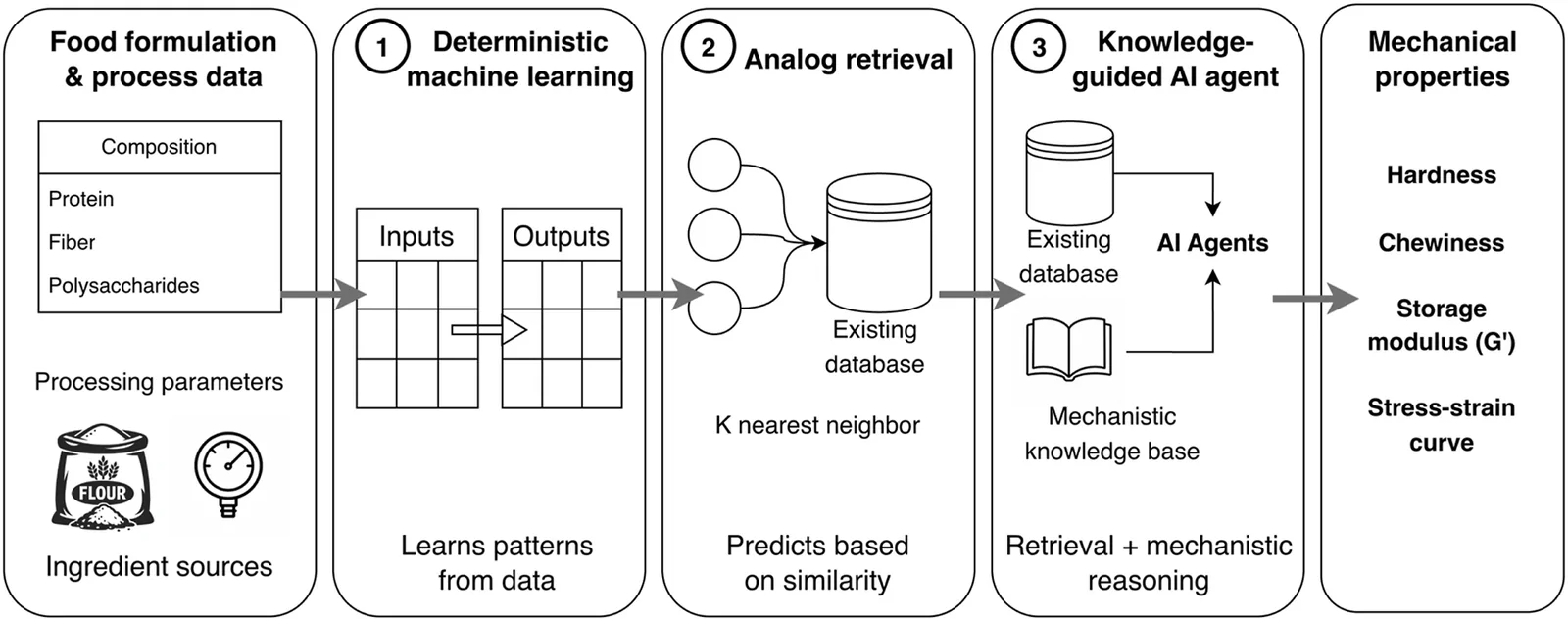

## 1 Introduction

Mechanical properties of food structures are co-determined by food composition, molecular interactions, water distribution, and processing history. In plant-based meat analogues, this complexity becomes especially pronounced because texture emerges from coupled phenomena such as protein denaturation, aggregation, phase separation, polysaccharide interactions, moisture redistribution, and shear-induced structuring during processing (Kyriakopoulou et al., 2021; Ishaq et al., 2022; Zahari et al., 2022). As a result, similar ingredient compositions can produce substantially different mechanical responses depending on ingredient source and process pathway. Understanding and predicting the texture of plant-based meat analogues therefore requires linking formulation, process, and structure rather than treating these products as simple compositional blends. It is also necessary to recognize the ingredient heterogeneity across botanical sources, extraction methods, and seasonal variations (Zhang et al., 2023; Fu et al., 2023; Vila-Clarà et al., 2024; Xie et al., 2024).

Machine learning (ML) has increasingly been used to address this challenge. In plant-based meat analogues, Kircali Ata et al. (2023) demonstrated that hardness and chewiness can be estimated from proximate composition using supervised learning, while Jiang et al. (2025) showed that machine-learning-assisted optimization can reduce experimental burden in high-moisture extrusion. Similar efforts have been reported for related food functionality problems, including hydrocolloid rheology in plant-based meat analogue formulations (Lee et al., 2024), rheological parameter prediction in plant protein-polysaccharide systems (Dahl et al., 2025), digestibility estimation from physicochemical descriptors (Liu et al., 2025), and texture perception from rheological descriptors (Kraessig et al., 2025). These studies demonstrated that data-driven models can be useful for food functionality predictions. However, their performance remains sensitive to dataset size, specific features, and distributional shift, which are recurring limitations in food research where experiments are expensive and sample numbers are small.

In parallel, artificial intelligence (AI) in food and nutrition has expanded from conventional ML to generative AI systems, with AI agents already applied to dietary assessment, food recognition, and nutrient intake estimation through computer vision and multimodal foundation models (Dalakleidi et al., 2022; Zheng et al., 2024; Lo et al., 2024). In this context, simple retrieval-based approaches such as inverse distance weighting (IDW) provide a baseline for leveraging similarity between known examples and making inference to new samples. More broadly, retrieval-augmented generation and tool-using AI agents have shown that predictions can be supported not only by data retrieved from examples, but also by external knowledge and explicit reasoning procedures (Yao et al., 2023; Schick et al., 2023). This shift is conceptually relevant to food science because functionality prediction often depends on both empirical analogues and mechanistic prior knowledge of ingredient functionality that may not be fully captured by a small numeric feature set.

Despite this promise, the use of knowledge-guided agents for food functionality prediction remains largely unexplored. This study compared deterministic ML, pure IDW retrieval, and a knowledge-based AI agent for predicting complex food structure functionality in two cases, namely plant-based meat analogue texture and plant protein-polysaccharide rheology. It was hypothesized that the AI agent would be most beneficial when test samples were out-of-distribution or when the target property depended on mechanistic distinctions that were only partly covered by the numeric inputs.

## 2 Materials and methods

### 2.1 Study design and datasets

Two held-out prediction tasks were assigned. The first task was based on the dataset of Kircali Ata et al. (2023), which contains 54 plant-based meat analogue samples characterized by seven compositional features, namely protein, fat, carbohydrate, fibre, ash, moisture, and target moisture, together with hardness and chewiness measurements. To create a stringent test, two sets of samples were held out as the test set, yielding 41 training samples and 13 test samples. This split was deliberately out-of-distribution because the held-out groups included formulations with fibre and protein levels outside the training range.

The second task was based on the dataset of Dahl et al. (2025), which contains 311 measurements on plant protein-polysaccharide mixtures with rheological outputs. After cleaning replicates by formulation, the data were split by formulations, yielding 246 training samples and 65 test samples; two structural outliers were excluded from the rheology evaluation, giving 63 test samples for the reported metrics. The two excluded samples reported maximum-stress values of roughly 4–7 Pa. This is about three orders of magnitude below the rest of the distribution and indicates measurement failure rather than genuine material behaviour. The exclusion was made on data-inspection grounds before running any predictions. The same 63-sample set was used identically for ML, IDW, and the agent. The prediction targets were storage modulus, $G^{\prime }$ [kPa], and maximum stress at 200% strain in the large deformation regime. The input features consisted of protein content, polysaccharide content, moisture content, and ingredient identity variables derived from protein source and polysaccharide source. The ML models, IDW, and the agent used the same input variables in each case. Any difference in information between methods is therefore limited to the mechanistic rules provided to the agent.

### 2.2 Prediction approaches

Gradient boosting (GB), random forest (RF), and TabPFN were applied as ML baselines, each trained exclusively on the training split with no access to test-set labels. GB and RF are ensembles of decision trees trained sequentially to minimise residual error and variance. TabPFN is a pre-trained transformer model that performs in-context learning by treating the labelled training set directly as input, without requiring iterative fitting, which makes it particularly well-suited to small-sample tabular regression. Gradient boosting hyperparameters (number of estimators, tree depth, and learning rate) were selected by grouped cross-validation grid search on the training split, ensuring no access to test-set labels. Random forest and TabPFN were used with their default settings, as these methods are relatively insensitive to tuning on small tabular datasets.

The second approach was pure IDW retrieval, which predicted each test sample from the k=3 nearest training samples in normalized input space. All pairwise distances were computed as the Euclidean norm after min-max normalization using training-set ranges, ensuring that no single feature dominated the distance metric due to scale differences. For a query sample $x^{*}$, the k nearest neighbours $xi{i=1}^{k}$ were identified, and the prediction was formed as

$$\hat{y}=\frac{\sum_{i=1}^{k}w_{i}\;y_{i}}{\sum_{i=1}^{k}w_{i}},\;w_{i}=\frac{1}{d_{i}},\;d_{i}={\left\|x^{*}-x_{i}\right\|}_{2}\text{,}$$

where $d_{i}$ is the normalized Euclidean distance between the query $x^{*}$ and the i-th neighbour $x_{i}$, and $y_{i}$ is the observed target value of the i-th neighbour. Exact matches ($d_{i}=0$), when present, were averaged directly to avoid division by zero. The choice of k=3 was fixed across both case studies to provide a stable low-variance baseline. Sensitivity of the retrieval baseline to the neighbourhood size was verified for k∈{1,2,3,5,7,10}, and k=3 was retained as a stable, low-variance setting. For the plant-based meat analogue case, IDW operated on seven numeric formulation variables. For the rheology case, IDW operated on three numeric composition variables, namely protein content, polysaccharide content, and moisture content, using formulation-averaged training data so that the retrieval base was identical to the agent setting.

The third approach was a knowledge-based AI agent, which used the same retrieval logic as IDW but incorporated explicit food-domain reasoning in the prediction step. Here, the term *agent* denotes a large language model that reaches a prediction by invoking external tools rather than by autonomous multi-step planning. In this study the model used two such tools, namely analog retrieval from the training set and a curated mechanistic knowledge base of food-structure rules. The agent operated in two stages. In the retrieval stage, the k=3 nearest training analogues were identified using the same normalized Euclidean distance as Equation [eq:idw], and their observed target values, input features, and contextual metadata were assembled into a structured prompt. In the reasoning stage, a large language model (Claude Sonnet 4.6, Anthropic) processed this prompt together with a compact mechanistic knowledge base encoded as task-specific skills. The mechanistic knowledge base consisted of two components: the structured training-set examples and a set of literature-derived mechanistic knowledge.

To construct knowledge base, for the plant-based meat analogue texture case, the agent combined analog retrieval with mechanistic priors on moisture-dependent protein network formation, the relative texturizing capacity of pea, soy, and wheat proteins under high-moisture extrusion, fibre-reinforced network stiffening, and fat-induced lubrication and softening (Kyriakopoulou et al., 2021; Ishaq et al., 2022; Zhang et al., 2023; Vila-Clarà et al., 2024). For the rheology case, the agent integrated retrieval with priors on concentration-dependent power-law gel strengthening ($G^{\prime }\propto c^{n}$), moisture-driven network dilution, protein-source ranking by gelation capacity, synergistic polysaccharide-protein co-network formation, and a brittleness correction for carrageenan-dominated systems at large deformation (Lee et al., 2024; Gao et al., 2024; Dahl et al., 2025). The brittleness rule, in particular, encoded the observation that κ-carrageenan gels exhibit brittle fracture under high strain that is not captured by small-deformation moduli, providing the agent with mechanistic context unavailable to composition-only ML models. The knowledge base was manually compiled from the cited literature and the training rows. Compilation was completed before any test-set prediction was run. Where the literature contained conflicting evidence, the rule was kept in qualitative form rather than as a numeric adjustment. In all cases, the test set remained inaccessible during model fitting and agent retrieval. Only input-side information was provided to the agent, namely the composition features and ingredient-source identifiers; no measured hardness, chewiness, $G^{\prime }$, or maximum-stress value of any test sample was included in the retrieval base or in the prompt, ensuring strict train–test separation. The mechanistic rules were derived from the published literature and from inspection of the training rows only. The agent was queried once per sample with a fixed structured prompt (Claude Sonnet 4.6; maximum output length 2048 tokens; sampling temperature = 1.0, the Anthropic API default). The complete prompt templates (task skills), the prediction-extraction code, and the per-sample model outputs are provided in the project repository. Because the retrieval step and the mechanistic rules are deterministic, the stochastic sampling of the language model affects mainly the language response rather than the numeric estimate. Numeric estimations remain stable, so run-to-run variation is therefore small relative to the differences between methods. The final numerical value is produced by the agent's own reasoning based on IDW-weighted average and mechanistic qualitative rules as context. The adjustment magnitude is chosen by the model within the qualitative range, so no closed-form correction factor is used. When no rule applies, the IDW-weighted average is emitted as the final answer. The detailed mechanistic knowledge base is available in the project repository (https://git.wur.nl/yizhou.ma/ml_vs_agent_texture_prediction).

### 2.3 Evaluation

Performance was assessed quantitatively using mean absolute error (MAE) and coefficient of determination ($R^{2}$). Bootstrap confidence intervals (95%) were computed for both metrics to evaluate the model stability. This evaluation framework allowed the effect of literature-synthesized knowledge to be examined explicitly by comparing pure retrieval against knowledge-augmented retrieval under the same train-test split.

### 2.4 Data and software availability

The processed datasets, machine learning scripts, inverse distance weighting (IDW) baseline, knowledge-agent skills, and evaluation outputs used in this study are available at: https://git.wur.nl/yizhou.ma/ml_vs_agent_texture_prediction.

## 3 Results and discussion

### 3.1 Plant-based meat analogue texture

The results for plant-based meat analogue texture are summarized in Table 1. For hardness, the knowledge-based agent achieved the lowest error, with MAE = 4.66 N and $R^{2}$ = 0.782, ahead of random forest, TabPFN, and pure IDW retrieval. Relative to the strongest ML baseline (gradient boosting), the agent was numerically better but statistically comparable: a paired bootstrap on the per-sample errors gave Δ MAE = 0.77 N (95% CI [−0.85, 2.35]), so the two methods cannot be distinguished on this small out-of-distribution test set. For chewiness, the agent and gradient boosting were likewise comparable (agent MAE = 4.45 J, $R^{2}$ = 0.696; gradient boosting MAE = 4.57 J, $R^{2}$ = 0.701; paired Δ MAE = 0.12 J, 95% CI [−1.48, 1.63]). In both targets, however, the agent significantly outperformed pure IDW retrieval (paired Δ MAE = 14.1 N for hardness and 11.2 J for chewiness, both 95% CIs excluding zero). In contrast, pure IDW retrieval performed poorly for both targets, with strongly negative $R^{2}$ values, showing that simply finding the nearest sample in normalized composition space alone was insufficient for robust prediction. The full comparison further showed that some purely statistical models were very unstable on this small and shifted test set, indicating that not all ML models were equally robust under extrapolative conditions.

**Table 1:** Prediction performance for plant-based meat analogue texture. Hardness (N) is the first compression peak force in texture profile analysis, whereas chewiness (J) is the mechanical energy required to chew the sample to a swallowable state; the two are distinct physical quantities derived from different parts of the same texture profile analysis curve. Mean absolute error and coefficient of determination are reported with 95% bootstrap confidence intervals.

Target	Method	Mean absolute error [95% CI]	Coefficient of determination ($R^{2}$) [95% CI]
Chewiness (J)	Agent	[2.86, 6.23]	[0.397, 0.811]
Chewiness (J)	IDW	[11.01, 20.59]	[-6.399, −1.206]
Chewiness (J)	Random forest	[5.15, 11.28]	[-1.020, 0.349]
Chewiness (J)	Gradient boosting	[3.03, 6.00]	[0.401, 0.818]
Chewiness (J)	TabPFN	[6.05, 12.98]	[-2.865, 0.430]
Hardness (N)	Agent	[3.43, 5.90]	[0.512, 0.869]
Hardness (N)	IDW	[13.21, 24.78]	[-7.731, −1.361]
Hardness (N)	Gradient boosting	[3.70, 7.12]	[0.112, 0.851]
Hardness (N)	Random forest	[3.73, 8.20]	[0.139, 0.755]
Hardness (N)	TabPFN	[11.36, 20.33]	[-6.739, −0.086]

Fig. 1 visualizes the hardness predictions. The contrast among gradient boosting, pure IDW, and the knowledge-based agent shows that the main benefit did not come from simple analog retrieval alone. The machine learning algorithm (Gradient boosting) behaved as a classifier as the predictions were grouped into 3 hardness levels across the hardness range (Fig. 1A). This is commonly reported for tree-based regression tasks, as they produce discrete outputs by assigning constant values within each split, rather than smooth continuous predictions. When predicting out of distribution samples, the pre-assigned split values failed to capture the variations in the test dataset. The pure IDW baseline, which used the same k=3 retrieval logic without mechanistic knowledge, failed substantially. Although it still produced numerical estimates, these were essentially uninformative (strongly negative $R^{2}$), likely due to the small training dataset and the out of distribution test set (Fig. 1B).

**Fig. 1:**
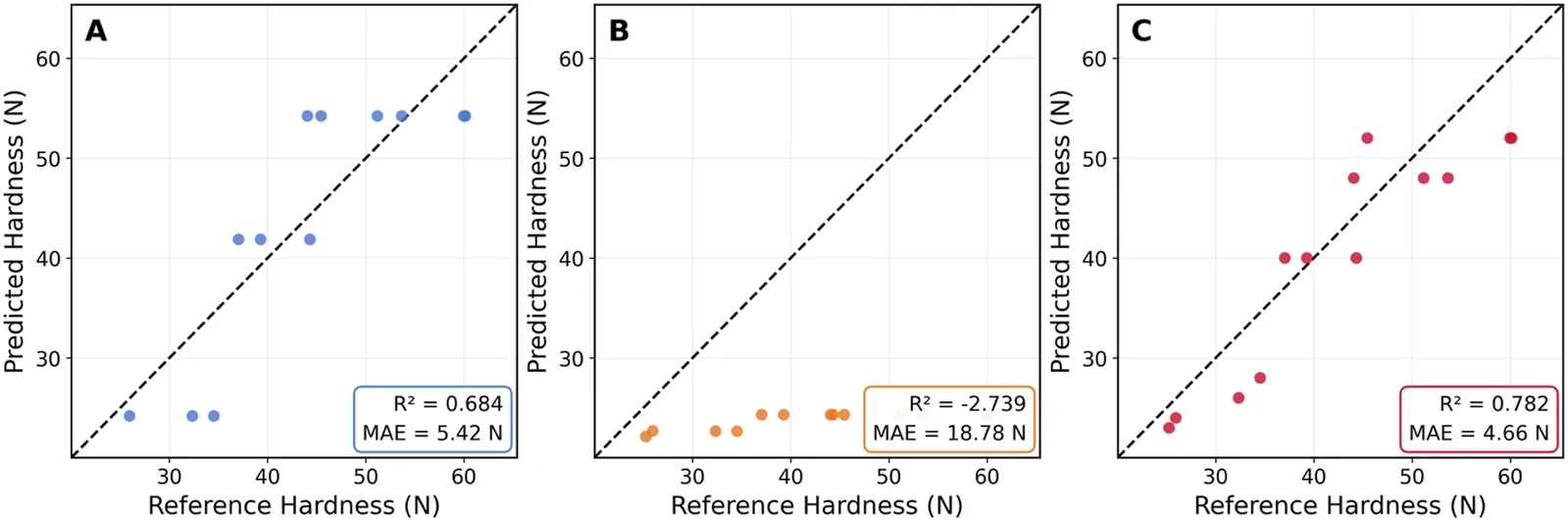
Predicted vs. reference hardness (N) for the out-of-distribution test set (Plant-based meat analogue texture, n=13). The dashed line indicates perfect prediction (x=y). (A) Gradient boosting (best-performing ML model): $R^{2}=0.684$, MAE = 5.42 N. (B) IDW (pure analog retrieval, k=3, no domain knowledge): $R^{2}=-2.739$, MAE = 18.78 N. (C) Agent (knowledge-augmented IDW, k=3): $R^{2}=0.782$, MAE = 4.66 N.

By contrast, the knowledge-based agent yielded predictions that were more tightly aligned with the parity line and modestly improved on the best ML baseline. The agent was able to handle a distribution shift in protein and fibre composition. Such distributional shifts are common in food formulation research, where new protein sources or altered fibre levels may be introduced. Under these conditions, the agent appears to have benefited from combining retrieved analogues with mechanistic rules related to target moisture, protein-network contribution, fibre reinforcement, and fat plasticization. That behavior is consistent with the broader understanding that plant-based meat analogue texture depends not only on proximate composition, but also on how water and biopolymer networks reorganize during processing (Kyriakopoulou et al., 2021; Zhang et al., 2023; Fu et al., 2023). In this case, knowledge retrieval therefore served as a way to stabilize prediction under shift, rather than merely as a replacement for deterministic ML models.

### 3.2 Protein-polysaccharide rheology

The results for protein-polysaccharide rheology are summarized in Table 2. Compared with the plant-based meat analogue texture case, the machine learning models performed more strongly, which is consistent with the larger dataset size available here (n=246). For $G^{\prime }$, all major ML baselines achieved moderate $R^{2}$ values, ranging from 0.507 to 0.633, and the pure IDW baseline remained useful, with $R^{2}=0.408$ and MAE = 237.06 kPa. The knowledge-based agent produced the best result, with MAE = 152.30 kPa and $R^{2}=0.695$, outperforming the best ML baseline (TabPFN, $R^{2}=0.633$) while also achieving a lower error. This suggests that larger datasets benefited all methods, but did not eliminate the advantage of knowledge retrieval.

**Table 2:** Prediction performance for protein-polysaccharide rheology. Mean absolute error and coefficient of determination are reported with 95% bootstrap confidence intervals (CI).

Target	Method	Mean absolute error [95% CI]	Coefficient of determination ($R^{2}$) [95% CI]
$G^{\prime }$ (kPa)	Agent	[26.04, 338.13]	[0.670, 0.877]
$G^{\prime }$ (kPa)	IDW	[60.82, 493.63]	[0.135, 0.445]
$G^{\prime }$ (kPa)	Gradient boosting	[37.38, 420.30]	[0.509, 0.749]
$G^{\prime }$ (kPa)	Random forest	[42.62, 439.86]	[0.322, 0.627]
$G^{\prime }$ (kPa)	TabPFN	[43.29, 385.15]	[0.361, 0.663]
Max stress 200% (kPa)	Agent	[11.77, 23.35]	[-0.005, 0.925]
Max stress 200% (kPa)	IDW	[17.58, 30.56]	[-0.196, 0.814]
Max stress 200% (kPa)	Gradient boosting	[28.34, 46.37]	[-1.460, 0.499]
Max stress 200% (kPa)	Random forest	[24.32, 40.37]	[-1.269, 0.699]
Max stress 200% (kPa)	TabPFN	[29.35, 49.40]	[-1.873, 0.427]

For maximum stress at 200% strain, the predictive performance among methods became more distinctive. The ML models showed the weakest performance, with MAE = 32.21 kPa and $R^{2}=0.412$, and predictions concentrated in a narrow band that failed to capture the full dynamic range of the test set. This reflects the tendency of ensemble tree models to regress toward the mean when the training signal is weak relative to target variability. This limitation is consistent with findings by Dahl et al., who reported that ML algorithms struggle to predict rheological properties in the large deformation regime, where input features based on composition and processing temperature are insufficient to capture complex protein-polysaccharide interactions. The pure IDW retrieval improved upon RF, achieving MAE = 23.65 kPa and $R^{2}=0.654$. As shown in Fig. 2B, it aligned reasonably well with the diagonal for mid-range samples but exhibited greater scatter at higher stress values, reflecting its reliance on geometric proximity in a feature space that does not explicitly encode large-deformation behavior.

**Fig. 2:**
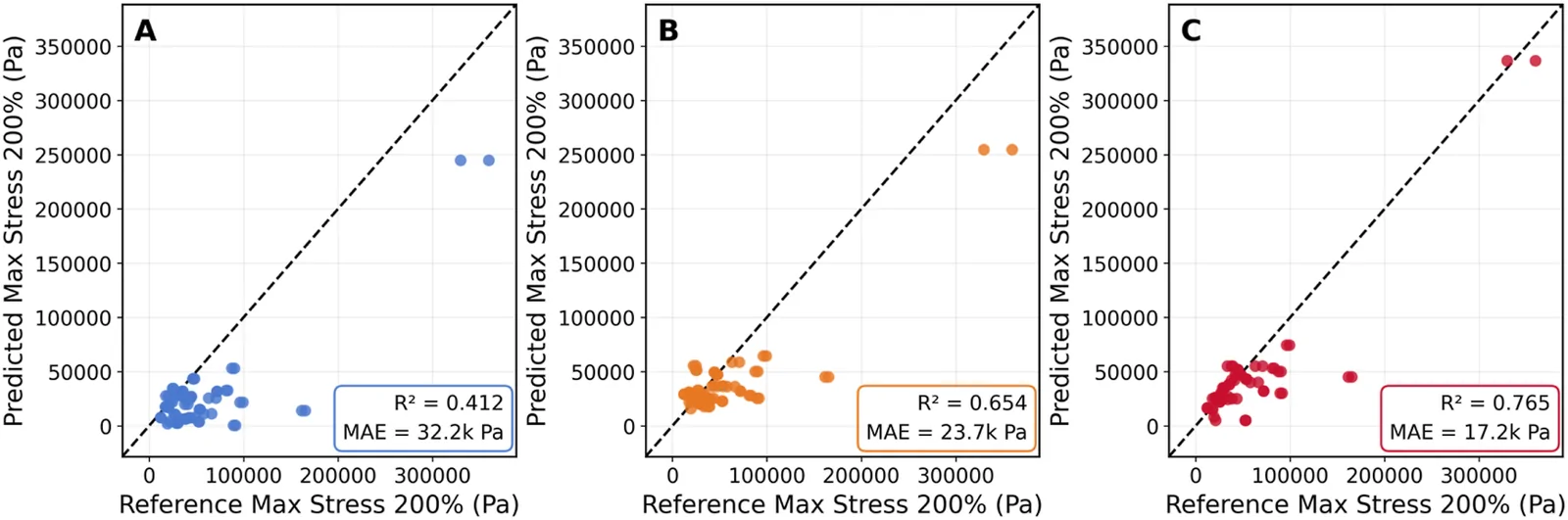
Predicted vs. reference maximum stress at 200% strain (kPa) for the held-out test set (biopolymer gel rheology, n=63, after excluding two structural outliers from the 65 held-out samples). The dashed line indicates perfect prediction (x=y). (A) RF (best-performing ML model): $R^{2}=0.412$, MAE = 32.21 kPa. (B) IDW (pure analog retrieval, k=3, no domain knowledge): $R^{2}=0.654$, MAE = 23.65 kPa. (C) Agent (knowledge-augmented IDW, k=3): $R^{2}=0.765$, MAE = 17.21 kPa.

The knowledge-based agent performed best, with MAE = 17.21 kPa and $R^{2}=0.765$. In Fig. 2C, its predictions followed the diagonal most closely across the measurable range, with a more symmetric distribution around the identity line. This indicates that incorporating mechanistic knowledge enables more accurate predictions, particularly at the extremes of the stress distribution where purely statistical approaches struggle. Overall, the predictive performance gap between methods is most pronounced under large deformation conditions. While increased sample size in the rheology dataset improved general model learning, the agent retained a clear advantage when the target property depended on mechanistic distinctions that were only partly captured by the available features.

### 3.3 General discussion

Across both cases, an important result is that pure IDW retrieval was not sufficient to achieve effective prediction compared to ML and knowledge-augmented agent. The predictive gain should therefore be attributed mainly to the integration of mechanistic knowledge with retrieved data, rather than to similarity-based retrieval alone. Consistent with this, preliminary tests in which the agent estimated each target from the retrieved analogues without the mechanistic knowledge base yielded results comparable to pure IDW retrieval, indicating that the improvement stemmed from the mechanistic knowledge rather than from the language model's general reasoning alone. The quantitative ablation is reported in Appendix Table A1. Removing the mechanistic knowledge base returned the agent's error to the IDW baseline in the out-of-distribution meat case. For maximum stress at 200% strain, the no-rules variant performed worse than IDW. The gain therefore comes from the encoded mechanistic knowledge rather than from the language model's general reasoning. This observation aligns with recent developments in language-model systems, where retrieval (data-driven) and reasoning (knowledge-augmented) are increasingly combined rather than used separately (Lewis et al., 2020; Yao et al., 2023; Schick et al., 2023). For food science, such a gray-box strategy is attractive because functionality is rarely characterized by data alone.

Another practical aspect is model stability. The bootstrap confidence intervals reported in Table 1, Table 2 reveal that prediction variance differed substantially across methods and dataset sizes. In the plant-based meat analogue texture case (n=13), confidence intervals were wide for all methods, reflecting the limited test set size rather than any intrinsic property of the predictors. In the rheology case (n=63), intervals narrowed considerably for $G^{\prime }$, with the agent achieving a relatively tight range of $R^{2}\in \left[0.670,0.877\right]$, compared with IDW ($R^{2}\in \left[0.135,0.445\right]$), indicating that knowledge-guided retrieval produced more stable estimates as dataset size increased. For maximum stress at 200% strain, confidence intervals remained wide across all methods, including the agent ($R^{2}\in \left[-0.005,0.925\right]$), which reflects the inherent difficulty of predicting large-deformation behavior from composition features alone. Taken together, the stability analysis suggests that the agent's advantage over IDW was consistent across both cases, and that its advantage over the ML baselines held in the rheology case, rather than being driven by favorable resampling outcomes, but that reliable uncertainty quantification for mechanically complex targets will require larger and more representative datasets.

The present study also contains some limitations. Only two case studies of tabular data were considered. The prediction targets were scalar values and did not involve kinetics or other physically meaningful outputs. For those cases, agent-based methods may suffer from maintaining physics consistency in predictions. Moreover, the quality of the agent depends on the encoded mechanistic knowledge. The present agent implementation was also not self-updating, meaning that new observations would need to be added manually to refresh the knowledge base. Future research should therefore investigate continuous learning frameworks in which the agent updates its knowledge base autonomously as new experimental observations become available, reducing the dependency on expert curation and improving adaptability over time. Additional case studies covering a broader range of food systems, processing conditions, and mechanical targets are also needed to establish the generalisability of knowledge-guided retrieval beyond the two cases examined in this study.

## 4 Conclusion

Knowledge-based AI retrieval was shown to be a viable approach for predicting food structure-related mechanical properties across two distinct cases, namely plant-based meat analogue texture and protein-polysaccharide rheology. In the plant-based meat analogue case, the main advantage of the agent appeared under out-of-distribution prediction, where mechanistic knowledge helped stabilize predictive performance well beyond pure similarity-based retrieval and on par with the best machine-learning baseline. In the rheology case, the larger dataset improved the performance of all methods, yet knowledge-guided retrieval still provided the best accuracy, particularly for the target associated with large-deformation failure behavior. Taken together, these findings show that explicit food-science knowledge can complement data-driven learning, and that retrieval augmented with mechanistic reasoning provides a practical route toward more robust prediction in small-data and partially observed food systems. This approach may therefore be useful for accelerating AI agent-based formulation screening and functionality prediction in plant-based food design and related structured food materials.

## Data availability

The data and modeling scripts developed in this study can be accessed at: https://git.wur.nl/yizhou.ma/ml_vs_agent_texture_prediction.

## CRediT author statement

Yizhou Ma: Conceptualization, Methodology, Data Curation, Formal Analysis, Writing – Original Draft.

## Declaration of generative AI technologies

During the preparation of this manuscript, AI-assisted tools were used for language refinement and Python scripting support. All scientific content, analysis, interpretation, and conclusions were developed and verified by the authors. The authors are fully responsible for the accuracy and integrity of the work.

## Declaration of competing interests

The authors declare that they have no known competing financial interests or personal relationships that could have appeared to influence the work reported in this paper.

## Figures and Tables

**Table A1:** Ablation results for the four prediction targets. Mean absolute error (MAE) is reported for three settings. The three settings are pure IDW retrieval, LLM with retrieval but without mechanistic rules, and the full knowledge-augmented agent. Units follow the corresponding target column.

Target	IDW	LLM + retrieval (no rules)	Full agent
Hardness (N)	18.78	18.76	4.66
Chewiness (J)	15.62	15.43	4.45
$G^{\prime }$ (kPa)	240.16	132.47	154.28
Max stress 200% (kPa)	23.89	30.65	17.38
